# Comparing two artificial intelligence software packages for normative brain volumetry in memory clinic imaging

**DOI:** 10.1007/s00234-022-02898-w

**Published:** 2022-01-15

**Authors:** Lara A. M. Zaki, Meike W. Vernooij, Marion Smits, Christine Tolman, Janne M. Papma, Jacob J. Visser, Rebecca M. E. Steketee

**Affiliations:** 1 Department of Radiology and Nuclear Medicine, Erasmus MC, University Medical Center Rotterdam, PO Box 2040, Rotterdam, 3000 CA the Netherlands; 2 Department of Epidemiology, Erasmus MC, University Medical Center Rotterdam, PO Box 2040, Rotterdam, 3000 CA the Netherlands; 3 Department of Neurology, Erasmus MC, University Medical Center Rotterdam, PO Box 2040, Rotterdam, 3000 CA the Netherlands

**Keywords:** Dementia, Magnetic resonance imaging, Atrophy, Diagnosis, Computer-assisted, Sensitivity and specificity

## Abstract

**Purpose:**

To compare two artificial intelligence software packages performing normative brain volumetry and explore whether they could differently impact dementia diagnostics in a clinical context.

**Methods:**

Sixty patients (20 Alzheimer’s disease, 20 frontotemporal dementia, 20 mild cognitive impairment) and 20 controls were included retrospectively. One MRI per subject was processed by software packages from two proprietary manufacturers, producing two quantitative reports per subject. Two neuroradiologists assigned forced-choice diagnoses using only the normative volumetry data in these reports. They classified the volumetric profile as “normal,” or “abnormal”, and if “abnormal,” they specified the most likely dementia subtype. Differences between the packages’ clinical impact were assessed by comparing (1) agreement between diagnoses based on software output; (2) diagnostic accuracy, sensitivity, and specificity; and (3) diagnostic confidence. Quantitative outputs were also compared to provide context to any diagnostic differences.

**Results:**

Diagnostic agreement between packages was moderate, for distinguishing normal and abnormal volumetry (K = .41–.43) and for specific diagnoses (K = .36–.38). However, each package yielded high *inter-observer* agreement when distinguishing normal and abnormal profiles (K = .73–.82). Accuracy, sensitivity, and specificity were not different between packages. Diagnostic confidence was different between packages for one rater. Whole brain intracranial volume output differed between software packages (10.73%, *p* < .001), and normative regional data interpreted for diagnosis correlated weakly to moderately (*r*_s_ = .12–.80).

**Conclusion:**

Different artificial intelligence software packages for quantitative normative assessment of brain MRI can produce distinct effects at the level of clinical interpretation. Clinics should not assume that different packages are interchangeable, thus recommending internal evaluation of packages before adoption.

**Supplementary Information:**

The online version contains supplementary material available at 10.1007/s00234-022-02898-w.

## Introduction

Dementia is a challenging clinical diagnosis based on multidisciplinary criteria. Magnetic resonance imaging (MRI) can aid the diagnosis of dementia as well as its subtype [1], as patterns of regional atrophy may indicate specific underlying pathology. However, visual assessment of these markers, for example using the MTA scale [2], can be sensitive to subjective evaluation, and is particularly challenging in early stages, when the visual profile is difficult to distinguish from healthy aging [3, 4]. Automated normative quantitative assessment of brain MRI implements algorithms to quantify patients’ regional brain volumetry, and compares them to data from a healthy reference population, potentially providing a more objective and reproducible method than visual assessment. Furthermore, use of normative quantitative information may improve earlier identification of atrophy [5, 6] and sensitivity and accuracy of radiological Alzheimer’s disease (AD) diagnosis [3, 7, 8].

Various proprietary artificial intelligence (AI) software packages that apply this quantitative method towards clinical use are emerging. However, understanding of its clinical implementation is limited [3, 4]. Most importantly, it is unclear whether these packages have equal and consistent advantages towards radiological diagnosis. Demonstrating consistent diagnostic contributions across different packages supports the efficacy of individual packages, as well as the validity of quantitative assessment for dementia diagnostics. Contrarily, any *differences* found between software packages and how they might influence the clinical process are important for clinicians to be aware of.

This study investigates two commercially available AI software packages, manufactured by Quantib B.V. and QUIBIM S.L., in a clinical setting. Specifically, the packages are compared in the context of neuroradiologists’ imaging-based diagnoses of dementia and its subtypes, to provide insight into whether different packages influence clinical radiological diagnosis in distinct ways. We explored potential differences between the softwares’ diagnostic contribution by assessing (1) agreement between diagnoses by neuroradiologists based on quantitative volumetric output; (2) diagnostic accuracy, sensitivity and specificity compared to the multidisciplinary clinical diagnosis; and (3) diagnostic confidence of neuroradiologists. To complement these assessments, we explored potential differences between the actual quantitative outputs produced by the two packages.

## Methods

### Participants

Patients who visited our memory clinic between 2010 and 2019, underwent MR imaging as part of their clinical work-up, and received a clinical diagnosis within six months of their MRI were eligible for this study. Diagnoses were based on multidisciplinary expert consensus including all available clinical, laboratory, and imaging data using standard diagnostic criteria for mild cognitive impairment (MCI), AD, and frontotemporal dementia (FTD) [9–12]. While MCI is a syndromal diagnosis and thus cannot be diagnosed based on imaging, this subgroup was included so as to represent the full spectrum of patients seen in the memory clinic.

Participants had to be ≥ 45 years old at time of MRI due to the lower age limit of the normative reference data of the software and have no record of comorbid neurological pathology. Following the exclusion criteria, 20 MCI patients remained, including 13 stable cases (no progression to dementia for at least 6 months following diagnosis) and 7 converters (progressed to dementia 6 months or later after diagnosis). To match proportions between diagnostic groups, 20 patients were randomly selected from the FTD group (13 behavioral and 7 language variant) and the AD group.

Twenty healthy controls were randomly selected from a set of 31 available from previous case–control studies [13, 14]. Healthy controls were included if they had no history of neurological complaints, which was subsequently verified by both full neuropsychological assessment and brain MRI. See Table 1 for sample demographics.

**Table 1:** Participant demographics

	AD (*n* = 20)	FTD (*n* = 20)	MCI (*n* = 20)	Control (*n* = 20)
Male (*n*)	10	12	14	12
Mean age in years (*SD*)	68.8 (7.4)	62.6 (6.9)	72.7 (7.4)	61.3 (6.6)
Age range in years	50–81	51–76	58–85	46–69

Age represents age in years at time of MRI scan

*n* number of subjects, *SD* standard deviation

All participants provided written informed consent. The institutional review board provided ethical approval for this study.

### Image acquisition

3D FSPGR T1-weighted MRI (GE Healthcare, USA) was acquired for all 80 subjects at 3.0 T (*n* = 67) or 1.5 T (*n* = 13) with isotropic (1mm^3^, *n* = 60) or near isotropic voxel acquisition size (~ 1 × 1 × 1.6mm^3^, zero-padded to 1 × 1 × 0.8mm^3^ for processing, *n* = 20).

### Software packages

The two software packages investigated in this study were the segmentation algorithms of Quantib® ND 1.5 software (Quantib, Rotterdam, Netherlands) for brain MRI analyses, and the QUIBIM Precision® Brain Atrophy Screening V1.0.0 module developed by QUIBIM S.L. (Quantitative Imaging Biomarkers in Medicine, Valencia, Spain). Quantib® ND’s segmentation algorithms include automated segmentation and quantification of brain tissue, cerebrospinal fluid (CSF), and brain structures (lobes, cerebellum, hippocampus) using T1-weighted scans and automated segmentation and quantification of white matter hyperintensities using T2-weighted FLAIR scans. For this study, only the segmentation and quantification of T1-weighted scans was used. QUIBIM Precision® Brain Atrophy Screening Analysis module automatically segments brain tissues (gray matter, white matter, and CSF) and parcellates the gray matter into 75 different regions and subregions from the frontal, temporal, parietal, and occipital lobes together with 15 additional subcortical structures. For the remainder of this paper, the packages from Quantib and QUIBIM will be referred to as Software 1 and Software 2, respectively.

Both packages use internally developed segmentation algorithms to produce absolute and relative regional brain volumes in a report that can be interpreted by neuroradiologists and integrated with PACS. Both packages implement a reference population to provide normative quantitative data that compares the individual patient’s volumetric data with that of a healthy population. Both software packages produce lateralized volumes for all lobes as percentage of intracranial volume (%ICV). Software 1 presents these lateralized lobar values in the report, while Software 2 includes %ICV of various lateralized substructures. For the comparative purpose of this study, Software 2 added the lateralized whole lobes (in addition to the substructures already present) to the report. See Table 2 and Supplementary Materials for details, as well as a recent review by Pemberton et al. [4] in which technical details and features of these and other software packages are summarized.

**Table 2:** Software package characteristics

	Software 1	Software 2
Certification	CE-marked and FDA cleared	CE-marked
%ICV values	%ICV represents gray and white matter combined	%ICV represents gray matter
Normative database	*N* = 4915; age range 45–95y; acquired at a single 1.5 T MRI	*N* = 620; age range 20–86y, extrapolated data for 87–90y; acquired at 1.5 T and 3 T MRI from 3 vendors
Normative data specificity	Subjects’ volumetry compared to age and sex-specific population	Subjects’ volumetry compared to age-specific population
Normative data presentation*	%ICV values are plotted on a reference curve for each structure, from which percentiles are deduced	Bar plots present relative %ICV values of each region against those of reference population

CE-marking = approval of medical device safety and performance and compliance with the EU Medical device legislation. Permission to market in the European Economic Area. FDA clearance = approval of medical device safety and effectiveness and permission to market in the USA by the US Food and Drug Administration

***%****ICV* percentage of total intracranial volume

*For examples of how the normative data is presented in each report, please see supplementary Fig. 1

### Procedure

A single brain MRI per subject was processed using both software packages to produce a quantitative report from each one, including information such as age, sex, regional volumetry, and its plotting against reference populations. All images were inspected for motion artefacts and other image distortions. Prior to processing, both software packages perform automated quality checks on required acquisition parameters. After processing, quantitative reports were checked for spurious results to identify potential segmentation errors.

These reports were provided to two neuroradiologist raters, blinded to clinical information and to the visual read of the MRI: one experienced, senior-level neuroradiologist (rater 1), and one neuroradiology fellow (rater 2). They each independently assigned a diagnosis to each report via a forced-choice design, without visual assessment of the actual images. Please note that this procedure is not according to intended use of either software package, which is to provide the trained medical professional with complementary information for the evaluation and assessment of MR brain images. We studied quantitative assessment only, as our aim was to compare software packages and the interpretation of their output reports, not to compare their performance or software-aided diagnoses.

First, raters indicated whether the volumetric profile was “normal,” or “abnormal” and then, *if abnormal*, whether the most likely diagnosis was “AD,” “FTD,” or “abnormal not classifiable.” MCI was not included as an option as it cannot be diagnosed based on imaging data. For each of these two steps, the neuroradiologists also rated their confidence in their diagnosis on a scale from one to five. Raters were not aware of the proportions of diagnostic groups in the sample, but were aware that it included subjects without a diagnosis of dementia, FTD patients, and AD patients. The raters first rated the Software 1 reports in one batch, then the Software 2 reports in one batch. Within each batch, reports were blinded to any patient information except age and sex, and presented in a different, randomized order.

### Statistical analysis

All diagnostic analyses were performed first for identifying dementia, i.e., whether the volumetric profile was “normal,” or “abnormal”, and then for specific diagnoses (normal, AD, FTD, or abnormal not classifiable). Statistical tests were performed using IBM SPSS Statistics 25, with a significance level of *p* ≤ 0.05.

#### Diagnostic agreement

We used Cohen’s Kappa (K) to evaluate (1) “inter-software agreement” of each subject’s diagnosis as assessed with each software package for each rater, and (2) inter-observer agreement between diagnoses as assessed by the two raters for each package.

Within each diagnostic group (AD, FTD, and controls), inter-software and inter-observer agreement was determined by simple agreement (proportion of identical diagnostic label, expressed in percentages) because sample sizes were too small for Cohen’s K analyses. All agreement analyses were performed irrespective of the accuracy of the diagnoses.

#### Diagnostic accuracy, sensitivity, and specificity

We measured accuracy, sensitivity, and specificity [15] using the multidisciplinary clinical diagnosis as the reference standard and compared these between the packages using McNemar tests. Since MCI is a syndromal diagnosis, imaging cannot indicate MCI specifically, implicating that an abnormal rating in MCI is technically a false positive, and a normal rating a false negative, as these patients are not clinically equivalent to healthy controls either. As these false positives/negatives affect accuracy, sensitivity, and specificity, these measures were evaluated without MCI patients.

#### Diagnostic confidence

Symmetrically distributed confidence data were analyzed using a Wilcoxon signed-rank test, otherwise using a sign test, for each neuroradiologist. These analyses were performed irrespective of the accuracy of the diagnoses.

#### Software quantitative output

Total brain matter %ICV values for each subject were compared between packages with a paired samples two-tailed *t*-test. Software 1 provides this *whole brain* %ICV value explicitly, and Software 2 provides the brain parenchyma fraction (which is equal to total brain matter %ICV). We also performed Spearman’s correlation analysis between regional normative data from Software 1 and from Software 2 (see Supplementary Materials).

## Results

### Diagnostic agreement

#### Dementia (normal versus abnormal volumetric profiles)

Inter-software agreement between Software 1-based labels and Software 2-based labels of normal and abnormal was moderate for both raters (K = 0.41, 95% CI [0.23;0.60], *p* < 0.001; K = 0.43, 95% CI [0.22;0.63], *p* < 0.001).

Software 1 exhibited good inter-observer agreement (K = 0.73, 95% CI [0.55;0.92], *p* < 0.001), and Software 2 very good inter-observer agreement (K = 0.82, 95% CI [0.69;0.94], *p* < 0.001).

#### Specific diagnoses (normal, AD, FTD, or abnormal not classifiable)

Both raters exhibited fair diagnostic agreement between their Software 1-based and their Software 2-based *specific diagnoses* (respectively K = 0.38, 95% CI [0.24;0.52], *p* < 0.001; K = 0.36, 95% CI [0.22;0.50], *p* < 0.001). For both raters, the highest inter-software agreement was found within the FTD group (95% for rater 1, 85% for rater 2). Lower inter-software agreement was found within the AD group (40% for rater 1, 45% for rater 2) and the control group (55% for rater 1, 50% for rater 2).

Inter-observer agreement for specific diagnosis of each subject was moderate, with K = 0.54, 95% CI [0.40;0.68] for Software 1 (*p* < 0.001), and K = 0.59, 95% CI [0.45;0.72] for Software 2 (*p* < 0.001). With Software 1, inter-observer agreement was highest among patients whose actual clinical diagnosis was FTD (90% simple agreement), followed by the control group (75%), then the AD group (55%). With Software 2, inter-observer agreement within FTD patients was also high (80%), but highest within the controls (90%). As with Software 1, inter-observer agreement within the AD group was lower (45%).

### Diagnostic accuracy, sensitivity, and specificity

The diagnostic accuracy, sensitivity, and specificity for the diagnosis of dementia (how accurately subjects were labeled as “normal” or “abnormal”), as well as for the diagnosis of AD and FTD specifically, are shown in Table 3. Sensitivity appears to be high for the diagnosis of dementia and FTD and lower for AD, but McNemar tests did not show significant differences in accuracy, sensitivity, and specificity between the packages.

**Table 3:** Diagnostic accuracy, sensitivity, and specificity with 95% confidence intervals for each rater, disease group, and software package

Rater	Disease	Software package	Accuracy (%)	Sensitivity (%)	Specificity (%)
1	Dementia	Software 1	85.0 (73.4; 92.9)	97.5 (86.8; 99.9)	60.0 (36.1; 80.9)
		Software 2	90.0 (79.5; 96.2)	90.0 (76.3; 97.2)	90.0 (68.3; 98.8)
	AD	Software 1	76.7 (64.0; 86.6)	50.0 (27.2; 72.8)	90.0 (76.3; 97.2)
		Software 2	78.3 (65.8; 87.9)	40.0 (19.1; 64.0)	97.5 (86.8; 99.9)
	FTD	Software 1	81.7 (69.6; 90.5)	95.0 (75.1; 99.9)	75.0 (58.8; 87.3)
		Software 2	86.7 (75.4; 94.1)	100.0 (83.2–100.0)	80.0 (64.4; 91.0)
2	Dementia	Software 1	83.3 (71.5; 91.7)	95.0 (83.1; 99.4)	60.0 (36.1; 80.9)
		Software 2	88.3 (77.4; 95.2)	90.0 (76.3; 97.2)	85.0 (62.1; 96.8)
	AD	Software 1	76.7 (64.0; 86.6)	50.0 (27.2; 72.8)	90.0 (76.3; 97.2)
		Software 2	75.0 (62.1; 85.3)	30.0 (11.9; 54.3)	97.5 (86.8; 99.9)
	FTD	Software 1	83.3 (71.5; 91.7)	95.0 (75.1; 99.9)	77.5 (61.6; 89.2)
		Software 2	81.7 (69.6; 90.5)	80.0 (56.3; 94.3)	82.5 (67.2; 92.7)

For the disease dementia, measures represent how well raters determined whether a subject’s volumetric profile was “normal” or “abnormal,” based on software report interpretation without visual assessment, and knowing that the sample consisted of subjects with and without dementia. For AD and FTD, measures represent how well raters determined whether or not a subject had that specific subtype. MCI patients excluded

*AD* Alzheimer’s, *FTD* frontotemporal dementia

### Diagnostic confidence

Wilcoxon signed-rank tests showed neuroradiologists’ diagnostic confidence was not different between software packages when distinguishing between normal and abnormal profiles (rater 1 (Mdn [IQR] = 5.0 [1.0] for both Software 1 and 2; rater 2 (4.0 [2.0] using Software 1 and 5.0 [1.0] using Software 2). We also compared the neuroradiologists’ confidence ratings in the *specific diagnoses* with a sign test, showing that rater 1 had significantly higher median confidence with Software 2 (4.0 [1.0]) than Software 1 (3.5 [2.0]) (*p* < *0.001*). For rater 2, median confidence was not different between Software 1 and 2 (4.0 [2.0] for both).

### Software quantitative output

**Fig. 1:**
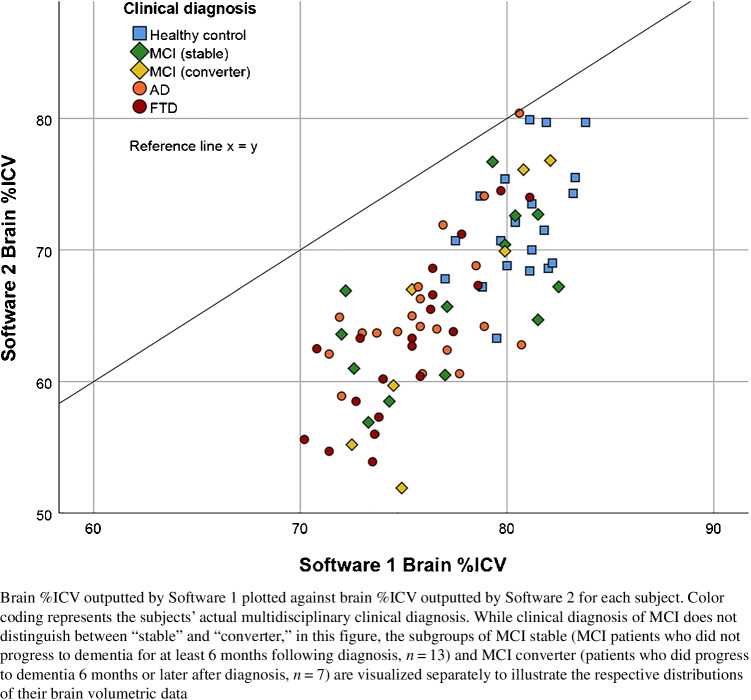
Correlation between Software 1 and Software 2 output of brain %ICV

Whole brain %ICV values outputted by Software 1 and Software 2 exhibited a significant difference of 10.73%, 95% CI (9.72; 11.75) (Software 1 [M = 77.28, SD = 3.54], Software 2 [M = 66.55, SD = 6.64]; *p* < 0.001). Regional normative data showed significantly positive correlations between packages (r_s_ values 0.27–0.80) for all regions, except the right occipital lobe (see Fig. 1 and Supplementary Materials).

## Discussion

We compared two commercially available normative volumetric quantification AI software packages to investigate their potential discrepant influences on dementia diagnosis in clinical practice. We found that agreement between software packages was moderate at most, when using quantitative reports in isolation to distinguish normal and abnormal profiles, or to make specific diagnoses. No significant differences were found in accuracy, sensitivity, and specificity between packages, but divergent patterns of diagnostic specificity were observed, as well as significant differences between total brain volume output.

Agreement between raters was high for each package when distinguishing normal and abnormal profiles. Moreover, with each package, both raters exhibited high accuracy and sensitivity when distinguishing normal and abnormal profiles. This suggests that normative quantitative data on its own has potential as an objective indicator of volumetric abnormality. This is less true for differential diagnoses, as inter-observer agreement of *specific diagnoses* was only moderate. This may reflect a lack of guidelines, as the software applications available for clinical practice, which are rapidly increasing, are consistently introduced without explicit instructions on how to interpret their results, and will likely be met with a learning curve for their use. At present, the lack of interpretation guidelines may lead clinicians to assume that different software packages perform similarly, and that they are interchangeable. Until now, this assumption has not been evaluated to the extent as has been done in this study. The differences observed here support the need for more studies of this kind, to develop the clinical guidelines required for optimal integration into practice.

While overall inter-observer agreement of specific diagnoses was moderate, inter-software and inter-observer simple agreement levels were higher for FTD than for AD and controls, as was sensitivity. This may indicate that atrophy patterns in FTD are more clearly reflected by quantitative assessment than in AD. Vernooij et al. found that adding quantitative to visual assessment did not improve the radiological diagnostic accuracy of FTD compared to visual assessment alone, while it did for AD [3]. Taken together, previous and current results suggest that while FTD may already be sensitively detected with quantitative information alone as well as with visual assessment alone, for AD diagnosis, the quantitative data alone is not quite as informative, but diagnosis can still be *enhanced* using the addition of quantitative information to visual assessment.

This study’s focus on clinical application of quantitative assessment tools is its most significant strength. Earlier studies have compared commercial algorithms for brain volumetry [16, 17] and also breast cancer [18], but to the best of our knowledge, this is the first study focused on *comparing commercial volumetric reports as they are interpreted* by radiologists in a memory clinic setting. Furthermore, there is strikingly limited research regarding the integration of these tools into clinical practice [3] and their clinical value—even FDA/CE clearance does not actually attest diagnostic efficacy [19, 20]. Although this study focused on the use of these packages in a *clinical context*, some software outputs were also compared, to provide insight into possible sources of diagnostic differences. The differences found between the packages’ total brain %ICV outputs indicate segmentation differences, but this is unlikely to explain diagnostic discrepancies as raters used *normative* data for their diagnoses. However, differences in reference populations and modest correlations between the packages’ normative data may have contributed (see Supplementary Materials). Additionally, the different visual presentation of normative data between packages may have influenced the diagnostic differences, including diagnostic confidence. In follow-up interviews, the more experienced rater reported a preference for the display of detailed substructures in Software 2 reports, while the fellow preferred the lobar reports from Software 1, which are more reflective of standard visual assessment (i.e., the Global Cortical Atrophy scale). Therefore, clinicians’ experience levels should be considered when choosing a package.

This study also has limitations, one being the use of quantitative reports without visual assessment of MRI, which does not reflect routine clinical practice, but did allow us to demonstrate that the output of different packages is interpreted differently, even in a forced-choice design. Using the quantitative reports in isolation operationalized our aim of comparing software packages to assess the consistency of diagnostic interpretations across different packages’ output, and not so much evaluate their performance or their relative contribution to software-aided diagnosis, which is a different field of endeavor. Additionally, the different graphical display of the reports made it impossible to blind the radiologists to the type of software.

Another limitation is that the results are specific to this study’s context, and are influenced by the packages used and the neuroradiologists’ experience. Results may therefore not translate to other clinics or packages. Nonetheless, the differences found in diagnostic agreement and specificity demonstrate that clinics cannot assume that the multiple software packages available are equally clinically efficacious *for them*. It would be particularly informative to expand the comparison and include more packages, and this need is increasingly being recognized by software companies as well. In a recently published review on technical and clinical validation of commercial automated volumetric MRI tools for dementia diagnosis, the majority of companies included in the review indicated that they would be willing to participate in a project comparing their reports and results, and their resulting clinical interpretation and impact [4]. Such future studies would provide more comprehensive insights into the influences of different packages on dementia diagnosis in clinical practice.

A final limitation is the composition and size of the diagnostic subgroups. First, the AD group included both early onset and late onset AD patients who have distinct imaging patterns on brain MRI and could therefore be considered as a separate groups. Still, we deemed adding both early and late onset AD patients as more representative for the entire memory clinic population. Moreover, the variability in imaging phenotype probably did not complicate the ratings, as raters had age information available through the quantitative reports, which helped them integrating the imaging abnormality profile in the report with a diagnosis of either late or early onset AD. Second, while inclusion of both MCI patients and healthy controls helped to reflect the true variety of patients seen in clinical practice, sample size was then limited by the exclusion of MCI patients from accuracy analyses. Third, we included diagnostic subgroups of equal size. Although we deemed this appropriate for the purpose of initial evaluation of the software packages, such an even distribution is not representative of the actual patient population of a memory clinic and may influence the parameters of diagnostic assessment.

In conclusion, this study presents initial evidence that different AI software packages that provide quantitative normative assessment of brain MR images can in fact produce distinct effects at the level of clinical interpretation. While individual quantitative assessment packages can potentially contribute to objective identification of dementia, we cannot assume that different packages can be used interchangeably. Future studies should explore whether these differences also exist between other packages and in other clinical settings, as well as work towards providing guidelines for the interpretation of quantitative normative volumetry in clinical practice. Memory clinics should be aware that choice of software package could impact diagnostic interpretation, also influenced by the characteristics of the clinic itself. Therefore, before adopting a specific package, evaluation *within* the specific clinic that wants to implement it is strongly suggested.

## Supplementary Information

Below is the link to the electronic supplementary material.

Supplementary file1 (DOCX 113 KB)
