# Supplementary material for: Comparing two artificial intelligence software packages for normative brain volumetry in memory clinic imaging
Source: Neuroradiology. 2022 Jan 15;64(7):1359–66. doi: 10.1007/s00234-022-02898-w (PMC9177657; doi:10.1007/s00234-022-02898-w)
Supplement: Supplementary file 1 — Supplementary file1 (DOCX 113 KB) [file 234_2022_2898_MOESM1_ESM.docx]

**Supplementary Materials**

**Methods**

**1. Reference populations**

The reference population that Software 1 uses is from the longitudinal population-based Rotterdam Study [1] and consists of 4915 people aged 45 to 95 years old whose scans were acquired using a 1.5T MRI system (GE Healthcare, US). The reference population used by Software 2 is composed of 620 Caucasian people aged 20 to 86 years old, with extrapolation of data from ages 87 to 90 years old. Their scans were acquired at both 1.5T and 3T systems from GE Healthcare, Siemens, and Philips (this information was acquired from personal correspondence with the vendors).


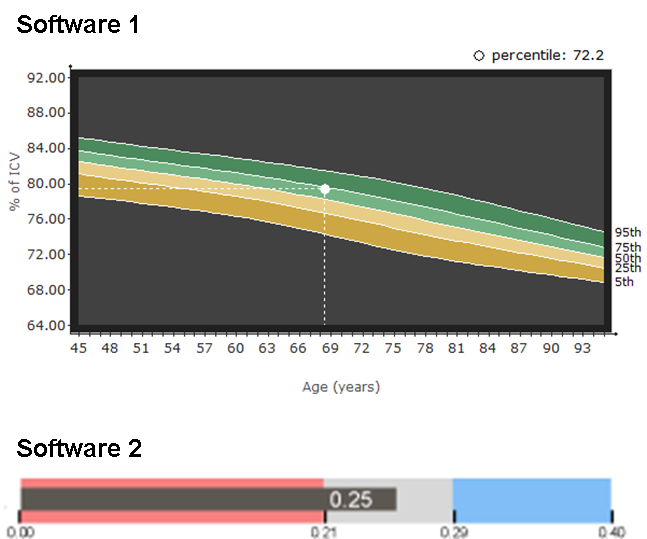


**Supplementary Fig. 1** Presentation of Normative Data in Output Reports.

Examples of visual representation of normative data output generated for the summary reports by Software 1 (top) and Software 2 (bottom) for arbitrary brain structures. Software 1 presents the volume (%ICV) versus age range (years). Reference curves, modeled after the volumes of the reference population, are plotted in the same graph. This enables deducing the percentile values from the graph. Software 2 presents %ICV values against those of the reference population of the patient’s age in a bar plot.

Examples are taken from sample reports that are either available for download ([Software 1 example](https://www.quantib.com/solutions/quantib-nd-plus)) or displayed directly ([Software 2 example](https://quibim.com/biomarker/atrophy-screening/)) on the vendor’s websites

**2. Correlation Analysis of Normative Output**

Normative data from the quantitative reports is what is most relevant to clinicians making diagnostic decisions. These data represent where each %ICV value falls relative to the healthy age-specific reference population. Software 1 presents regional normative data in the quantitative report in the form of %ICV values that are plotted on a reference curve, as well as percentile values deduced from this. Software 2 presents normative data by plotting each regional %ICV value against those of the reference population visually in bar plots. Therefore, we did not have quantified normative data from Software 2. In order to assess the correlation between the packages’ normative data, we converted the %ICV values from the Software 2 reports into *z*-scores that represent where they fall relative to the healthy age-specific population. The conversion was based on the mean and standard deviation of the age-specific and region-specific reference population from Software 2 for each %ICV data point. A Spearman’s correlation was performed, as the data did not meet the normality assumptions of the Pearson’s correlation. The analysis was performed separately for each lateralized lobe, and lateralized hippocampi.

There were some missing percentile values from Software 1, as on some occasions the patient’s %ICV was beyond the data available in the corresponding reference population. These are not true missing values; they represent extreme cases that should be reflected in the analysis in order to see how well they correlate with results from Software 2. Therefore, we replaced these missing values with new values. For the cases in which the %ICV was too high for the percentile range, we assigned the value 99.99. Since Software 1’s percentiles have one decimal place, using 99.99 reflects that this value is higher than the rest, but still reflects the unit of percentiles. For the cases in which the %ICV was too low for the percentile range, we assigned the value 0.01, This method likely caused Software 1’s percentile distributions to be not normal, but performing a Spearman’s correlation accommodated this.

**References**1. Ikram MA, van der Lugt A, Niessen WJ, Koudstaal PJ, Krestin GP, Hofman A et al. (2015) The Rotterdam Scan Study: design update 2016 and main findings. Eur J Epidemiol 30:1299–1315.

**Results**

Supplementary Table 1 shows the results of Spearman’s correlations between packages for each region tested.

| Brain Region | *r*_s_ (78) | *p* |
| --- | --- | --- |
| Right Frontal Lobe | **.68** | **<.001** |
| Left Frontal Lobe | **.65** | **<.001** |
| Right Parietal Lobe | **.27** | **.017** |
| Left Parietal Lobe | **.36** | **.001** |
| Right Occipital Lobe | .12 | .280 |
| Left Occipital Lobe | **.35** | **.002** |
| Right Temporal Lobe | **.66** | **<.001** |
| Left Temporal Lobe | **.80** | **<.001** |
| Right Hippocampus | **.64** | **<.001** |
| Left Hippocampus | **.70** | **<.001** |

**Supplementary Table 1** Spearman’s Correlation between Normative %ICV Data Produced by Softwares 1 and 2.

Normative %ICV Data = Data representing where the region’s percentage of ICV falls, when compared to an age-specific reference population; for Software 1 in the form of percentiles, and for Software 2 in the form of *z*-scores. The percentiles were produced directly by Software 1. The z-scores were not produced directly by Software 2, but were calculated internally based on reference-population data from Software 2 for each data point. *r*_s_(78) = Spearman’s correlation coefficient, with degrees of freedom equal to 78 (*N*-2). Difference is significant at the 0.05 level.
